# Intrauterine volvulus: systemic review of the literature with pooled analysis

**DOI:** 10.1038/s41372-024-01984-6

**Published:** 2024-05-04

**Authors:** Tuğba Acer-Demir, Nihal Şahin-Uysal

**Affiliations:** 1 Department of Pediatric Surgery, Başkent University, Faculty of Medicine, Ankara, Turkey; 2 Department of Obstetrics and Gynaecology, Başkent University, Faculty of Medicine, Ankara, Turkey

**Keywords:** Intestinal diseases, Risk factors

## Abstract

Our objective is to analyse the observations related to intrauterine volvulus and assess how clinical manifestations and treatment strategies impact prognosis. We conducted a comprehensive search on Pubmed and ClinicalTrials.gov from inception to July 2022, using search terms like “intrauterine volvulus” or “foetal volvulus,” supplemented by manual scrutiny of reference lists in relevant texts and articles. Our review encompassed 57 case reports/case series, involving 88 cases. The presence of foetal bradycardia during prenatal visits (*p* = 0.002) and the existence of meconium cyst or pseudocyst (*p* = 0.038) significantly influence survival rates. Preterm labour occurred more frequently among cases resulting in mortality (54% vs 21%; *p* = 0.055). Our study’s limitations include the inability to access all reported cases and reliance solely on available data. We advocate for vigilant monitoring of foetuses exhibiting signs of intestinal obstruction, and consideration of an emergent caesarean section as a pre-emptive measure before foetal biophysical profile deterioration worsens.

## Introduction

Midgut or intestinal volvulus arises when loops of the intestine become twisted around the pedicle of the superior mesenteric artery. This condition constitutes a surgical emergency due to the heightened risk of mortality and morbidity associated with delayed diagnosis and treatment [1]. Although the volvulus is mostly observed during the infant period, intrauterine volvulus cases were also reported [2].

The causes of postnatal volvulus are mostly malrotation, followed by cystic fibrosis and gastrointestinal mass [1]. The majority of intrauterine volvulus cases occur without any predisposing factors. Less commonly, intrauterine volvulus can be attributed to conditions such as malrotation, intestinal atresia, meconium ileus (associated with cystic fibrosis), or anomalies in the mesentery [3]. For most cases, signs of intrauterine volvulus are non-specific; thus, intrauterine intestinal volvulus, a very rare life-threatening condition, is difficult to diagnose prenatally [4, 5].

We review case reports and series regarding intrauterine volvulus in English literature with the aim of identifying diagnostic indicators and assessing how clinical findings and treatment methods influence prognosis.

## Methods

### Search

We conducted a thorough search on PubMed (http://www.pubmed.ncbi.nlm.nih.gov) and ClinicalTrials.gov (http://www.clinicaltrials.gov) spanning from their inception to July 2022. Our search utilised the terms “intrauterine volvulus,” yielding 91 results on PubMed, and “foetal volvulus,” yielding 295 results on PubMed and 4 results on ClinicalTrials.com, all pertaining to English literature.

### Study selection

**Fig. 1:**
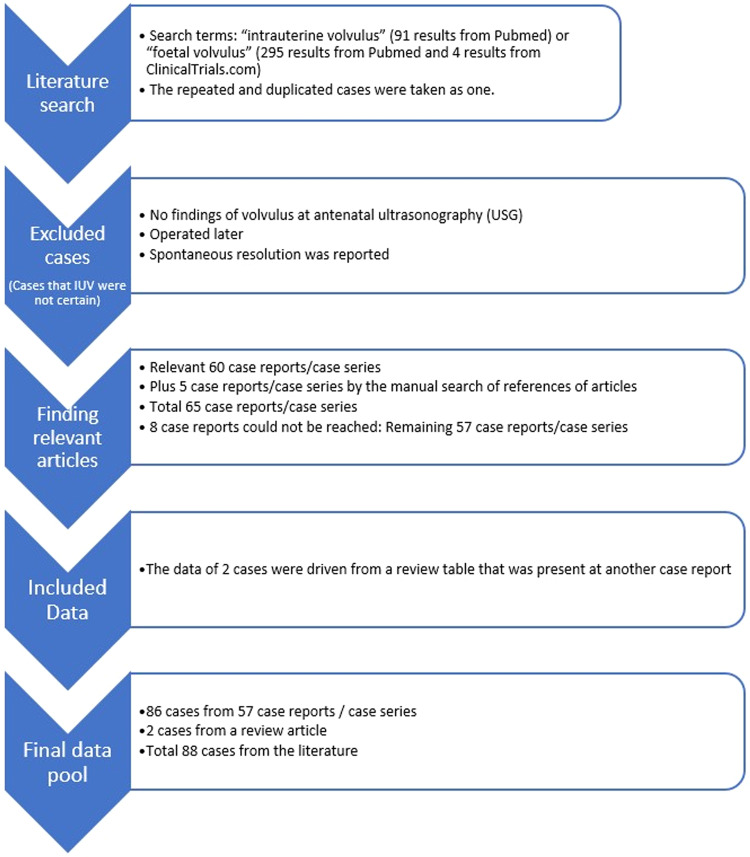
Literature review with included and excluded cases.

The article headings were scrutinised, and potential case reports and case series concerning intrauterine volvulus were identified. Instances of repetition or duplication were treated as singular cases. The abstracts of these articles were then examined to ascertain whether they indeed reported cases of intrauterine volvulus. We selected case reports or case series with headings explicitly indicating a focus on intrauterine volvulus, where the cases were presented as instances of intrauterine volvulus with either prenatal diagnosis via prenatal ultrasonography or operative findings immediately after birth. In total, we identified 60 case reports or case series. Unfortunately, access to 8 of these articles was not possible. However, we included 2 cases reported in detail in another case review. [6] We reached 52 articles, read them and list the findings of cases to a table for analysis (dataset). Additionally, we manually examined the reference lists of the included texts and relevant articles to identify any cases of intrauterine volvulus not captured in the initial literature search. This method led to the discovery of four case reports and one case series. These findings were then incorporated into the dataset. In conclusion, we conducted a review of 57 case reports or case series, providing adequate information on a total of 88 cases (as depicted in Fig. 1 and dataset) [1–57].

We omitted cases lacking volvulus findings on antenatal ultrasonography (USG) and those not promptly operated on after birth (presumed to be postnatal volvulus), as well as those documented to have undergone spontaneous resolution (specifically, three cases from the case series by Bartholmot et al.) [1].

### Data İtems

One reviewer (TAD) meticulously recorded the information of these cases in a table as they were reported, leaving unreported data blank in the table. Subsequently, we conducted statistical analysis on these data. The recorded data encompassed various parameters, including antenatal USG findings, foetal biometry and biophysical profile scores, gestational age at diagnosis and birth, birth weight, gender, physical examination results, radiological and operative findings of the newborn, type of surgical procedure performed (whether anastomosis or enterostomy), associated pathologies, potential aetiological factors of volvulus, and outcomes. The duration of volvulus was determined by subtracting the gestational age at diagnosis from the gestational age at birth.

In cases where certain features or findings were not reported, we assumed them to be within normal range. For prenatal diagnosis of the pathology, any suspicious prenatal ultrasound findings such as the presence of dilated bowel or ascites were accepted, influencing both prenatal and postnatal diagnoses accordingly.

Although necropsy findings from four cases reported by Ashworth [57] were documented in the dataset, they were not included in the operative findings, which were noted based on explorative laparotomies conducted after birth.

In instances where “no signal within/between loops” was reported on Doppler Ultrasound, this was defined as “Abnormal Doppler Findings.” Given that most authors did not specify whether bowel atresia occurred in the small bowel or large bowel, the term “bowel atresia” was used generally.

### Synthesis of results and statistics

We compiled the data into a table (dataset) and conducted statistical analysis. Cases were grouped based on survival outcomes: Group 1 included survived cases, while Group 2 encompassed lost cases, including miscarriage, intrauterine death, and postnatal loss cases. Four cases underwent pregnancy termination, and five cases lacked outcome data; hence, these cases were excluded from grouping.

Statistical analyses were performed using the IBM SPSS Statistics Version 25.0 software program (IBM Corp., Armonk, NY, USA). Descriptive statistics for categorical variables were presented as numbers (n) and percentages (%). For numerical parameters, if parametric test assumptions were met, descriptive statistics were presented as mean ± standard deviation. If parametric test assumptions were not met, the median and minimum-maximum values were reported. Parametric continuous variables were compared using the t-test, while non-parametric continuous variables were compared using the Mann–Whitney U test. Categorical variables were compared using either the Pearson chi-square test or Fisher’s exact test. A *p* value of less than 0.05 was considered statistically significant.

## Results

**Fig. 2:**
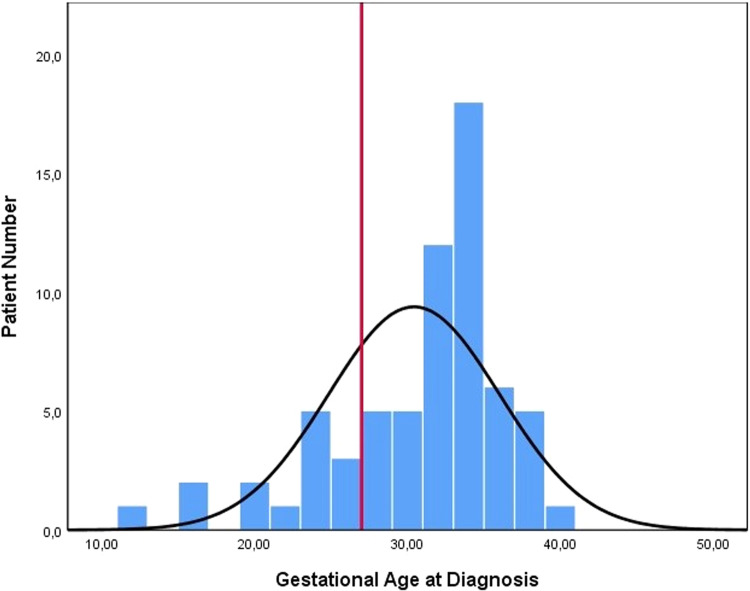
Distribution of gestational age at diagnosis (line: 26th gestational week).

**Fig. 3:**
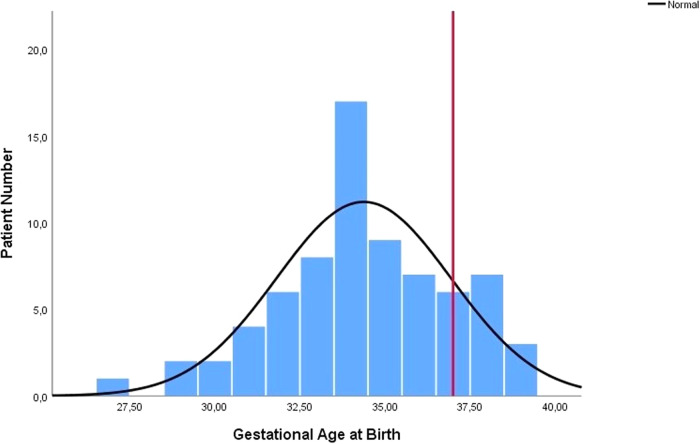
Distribution of gestational age at birth (line: 37th gestational week).

**Fig. 4:**
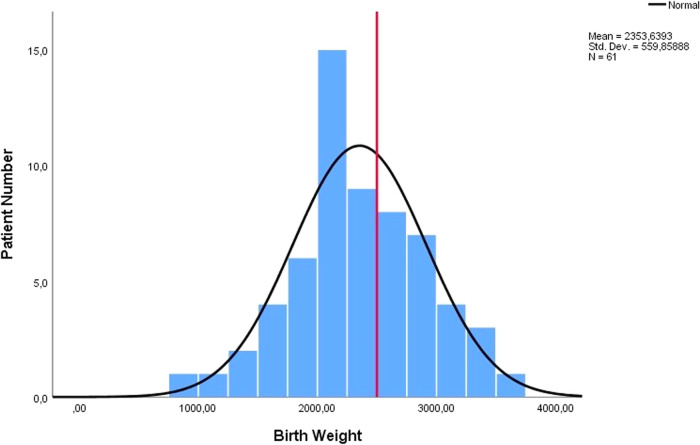
Distribution of birth weight (line: 2500 gr).

We reviewed 57 case reports or case series containing enough information on 88 cases (Fig. 1, dataset) [1–57]. Tables 1, 2 present the distribution of cases and provide the *p*-values for the comparison between Groups 1 and 2 (Tables 1, 2). Table 3 displays the distribution of gestational age at diagnosis and birth, birth weight, and duration of volvulus, along with the corresponding *p* values for each finding in the comparison between Groups 1 and 2 (Table 3; Figs. 2–4).

**Table 1:** Distribution of gender of cases, possible aetiological causes, operation, birth, and outcome.

Features	Total Patient (%)	Group 1 Patient (%)	Group 2 Patient (%)	*P* value
Gender				1.000
Male	32 (50%)	25 (49%)	6 (50%)	
Female	32 (50%)	26 (51%)	6 (50%)	
Diagnosis Time				0.002
Prenatal	69 (78%)	53 (84%)	7 (44%)	
Postnatal	19 (22%)	10 (16%)	9 (56%)	
Pregnancy				1.000*
Single	76 (90%)	56 (89%)	15 (94%)	
Twin	7 (9%)	6 (9%)	1 (6%)	
Triplet	1 (1%)	1 (2%)	0 (0%)	
Possible Aetiological Factor of Volvulus				0.047**
None	39 (48%)	27 (44%)	12 (67%)	
Atresia	22 (29%)	17 (28%)	3 (17%)	
Malrotation	13 (17%)	11 (18%)	2 (11%)	
Meconium Ileus	10 (13%)	8 (13%)	0 (0%)	
Anomaly of Mesentery	5 (6%)	4 (7%)	1 (5%)	
Operation				0.653
Anastomosis	41 (68%)	37 (69%)	3 (60%)	
Enterostomy (jejonostomy. ileostomy. vs)	19 (32%)	17 (31%)	2 (40%)	
Newborn				0.167
Preterm***	56 (76%)	49 (78%)	7 (58%)	
Term	18 (24%)	14 (22%)	5 (42%)	
Obstetric outcomes				
Spontaneous Preterm Labour	14 (21%)	9 (16%)	4 (40%)	
Urgent Caesarean Delivery (preterm)	27 (40%)	24 (42%)	3 (30%)	
Urgent Caesarean Delivery (term)	3 (4%)	3 (5%)	–	
Planned Delivery (Caesarean Section or Vaginally) (preterm)	10 (15%)	10 (18%)	–	
Planned Delivery (Caesarean Section or Vaginally) (term)	14 (20%)	11 (19%)	3 (30%)	
Outcome				
Lost	13 (20%)			
Survived	63 (80%)			
Intrauterine Death	3 (4%)			
Pregnancy Terminated	4 (5%)			

(Group 1 survived cases and Group 2 lost cases. Nine cases were not included from the grouping: four cases underwent pregnancy termination, and five cases lacked outcome data. Additionally, some reports contained missing data. As a result, disparities exist between the sum of groups and the total results reported, as well as between the number of cases and the total number reported).

^*^: *p* value calculated according to a single pregnancy or multiple pregnancies.

**: *p* value calculated according to the presence or absence of aetiological factors.

***: The cases with miscarriages were not included.

**Table 2:** Distribution of findings (antenatal ultrasound, biophysical profile, physical and radiological examinations, operative) and associated neonatal morbidities.

Findings	Total patient (%)	Group 1 patient (%)	Group 2 patient (%)	*P* value
Prenatal ultrasound findings				
Dilated Bowel	57 (72%)	46 (%75)	4 (44%)	0.107
Ascites	21 (27%)	15 (25%)	3 (33%)	0.685
Polyhydramnios	20 (25%)	17 (28%)	2 (22%)	1.000
Whirlpool Sign	19 (24%)	15 (25%)	2 (22%)	1.000
Abdominal Distention	14 (18%)	14 (23%)	0 (0%)	0.188
Abdominal Mass	12 (15%)	8 (13%)	2 (22%)	0.607
Meconium Peritonitis	11 (14%)	8 (13%)	2 (22%)	0.607
Pseudocyst	7 (9%)	6 (10%)	1 (11%)	1.000
Abnormal Doppler Findings	5 (6%)	4 (7%)	1 (11%)	0.508
Dilated Stomach	5 (6%)	5 (8%)	0 (0%)	1.000
Foetal Wellbeing				
Decreased Foetal Movements	28 (48%)	23 (51%)	3 (33%)	0.470
Non-Reassuring Foetal Testing	21 (41%)	18 (41%)	3 (43%)	1.000
Decreased Short Term Variability	15 (28%)	14 (31%)	1 (7%)	0.417
Findings of Foetal Anaemia	6 (10%)	5 (11%)	1 (12%)	1.000
Foetal Bradycardia	**5 (9%)**	**1 (2%)**	**4 (44%)**	**0.002**
Obstetric Outcomes				
Emergency Caesarean	30 (42%)	27 (47%)	3 (27%)	0.323
Preterm Labour	19 (27%)	12 (21%)	6 (54%)	0.055
Physical Examination Findings				
Abdominal Distention	46 (90%)	37 (90%)	8 (89%)	1.000
Cullen’s sign	17 (33%)	13 (32%)	4 (44%)	0.467
Bilious Gastric Aspirate	13 (26%)	11 (27%)	1 (11%)	0.425
Tender Abdomen	10 (20%)	10 (24%)	0 (0%)	0.174
Neonatal Abdominal Radiograph*				
Gasless Abdomen except Stomach	9 (27%)	8 (29%)	1 (20%)	
Dilated Bowel	8 (24%)	7 (25%)	1 (20%)	
Gasless Abdomen	5 (15%)	4 (14%)	1 (20%)	
Air-Fluid Levels	5 (15%)	3 (11%)	2 (40%)	
Neonatal Abdominal Ultrasound*				
Dilated Bowel	8 (57%)	7 (58%)	1 (50%)	
Abdominal Mass	4 (29%)	3 (25%)	1 (50%)	
Operative Findings				
Volvulus	78 (95%)	59 (97%)	10 (83%)	0.124
Necrosis	40 (56%)	31 (57%)	8 (73%)	0.503
Bowel Atresia	18 (22%)	14 (23%)	3 (25%)	1.000
Perforation	16 (22%)	13 (24%)	2 (18%)	1.000
Peritonitis	12 (17%)	11 (20%)	0 (0%)	0.187
Meconium Cyst/Pseudocyst	**9 (12%)**	**5 (9%)**	**4 (36%)**	**0.038**
Dilated Bowel	8 (11%)	7 (13%)	1 (9%)	1.000
Ascites	7(10%)	7 (13%)	0 (0%)	0.592
Hemoperitonium	6 (8%)	5 (9%)	1 (9%)	1.000
Associated Neonatal Morbidity				
Intubation and Ventilation	12 (19%)	8 (15%)	4 (36%)	0.197
Short Bowel Syndrome	11 (17%)	8(15%)	2 (18%)	1.000

(Group 1 comprises survived cases, while Group 2 consists of lost cases. Nine cases were not included in the grouping: four cases underwent pregnancy termination, and five cases lacked outcome data. Additionally, some reports contained missing data. Consequently, there are discrepancies between the sum of groups and the total results reported, as well as between the number of cases and the total number reported).

^*^: Statistical analysis could not be performed for certain parameters due to the small number of cases, which rendered meaningful statistical comparisons unfeasible.

Abnormal Doppler Findings: No signal within/between loops at Doppler Ultrasound.

Foetal Anaemia: The cases that were reported to have ‘foetal anaemia’ by the author (Hct 20-32%; Hb:7.1–9.9 g/dL).

Bowel Atresia: The distinction between small bowel and large bowel involvement was not specified in the reports.

**Table 3:** Distribution of gestational age at diagnosis and birth, birth weight, and duration of volvulus.

Data Type	Total	Group 1	Group 2	*P* value
Gestational age at diagnosis	32 weeks (12–39 weeks)	32 weeks (12–39 weeks)	32 weeks (24–35 weeks)	0.811
Gestational age at birth*	34 weeks (27–39 weeks)	34 weeks (27–39 weeks)	34 weeks (30–39 weeks)	0.947
Birth weight	2353.6 ± 559.9 gr	2322.4 ± 557.8 gr	2461.5 ± 593.0 gr	0.479
Duration of volvulus	1 week (0–16 weeks)	1 week (0–15 weeks)	0 week (0–13 weeks)	0.680

(Group 1 survived cases and Group 2 lost cases).

^*^: Cases with miscarriage were not included.

Our analysis revealed significant differences between Groups 1 and 2. Specifically, the presence of foetal bradycardia at prenatal visits (*p* = 0.002) and the presence of meconium cyst or pseudocyst reported during the operation (*p* = 0.038) were notably lower in Group 1 compared to Group 2. Most cases in Group 1 (84%) were diagnosed prenatally, a statistically significant difference from Group 2 (44%) (*p* = 0.002) (Table 1). Among the five cases reported to have foetal bradycardia, four cases resulted in mortality. Additionally, among the three cases with foetal hydrops, only one survived while two died.

Although preterm labour was more frequent in Group 2 (54% vs. 21%; *p* = 0.055), this difference did not reach statistical significance. Among the cases with preterm labour, two out of five delivered via caesarean section, and four out of twelve delivered vaginally resulted in mortality. In Group 1, the proportion of term newborns was 22%, while in Group 2, it was 42% (*p* = 0.167). Among the cases, three out of 27 delivered via emergent caesarean section, four out of 13 with preterm birth without urgent caesarean section, and four out of 25 with planned delivery resulted in mortality. However, there was no statistically significant difference between urgent caesarean section and planned delivery (*p* = 0.689) or between preterm birth without urgent caesarean section and urgent caesarean section (*p* = 0.172).

Among the 67 cases with reported antenatal diagnosis time, 79% were diagnosed after 27 weeks of gestational age (Fig. 2). Regarding preterm births, 55.6% occurred before 34 weeks gestation, 30.6% were late preterm births, and 13.9% were term births.

Furthermore, additional physical examinations of three cases were reported as normal. Notably, in one of these cases, although intrauterine death occurred at the 39th gestational week, the postnatal physical examination was normal [28].

Additionally, contrast enema was conducted for six cases, yielding findings such as narrow or microcolon (3 cases), normal colon (2 cases), caecum at the right upper quadrant (1 case), and suspected stenosis at the terminal ileum (1 case). Upper gastrointestinal series were performed for six cases, resulting in reports of normal findings (3 cases), obstruction at mid-jejunum (1 case), malrotation (1 case), and barium stopping at the level of the right lower quadrant, along with a “bird beak” sign with a filling defect (1 case).

Operative findings were available for 82 cases, with four of these cases not explicitly mentioning volvulus; however, they were reported to have pseudocyst/ meconium cyst secondary to volvulus [8, 9, 14, 56]. Four of 11 cases with pseudocyst/ meconium cyst, and seven of 49 cases without pseudocyst/meconium cyst died (*p* = 0.038).

Fifteen cases were found to be associated with foetal anaemia. Among these, six out of eight cases with hemoperitoneum and nine out of 56 cases without hemoperitoneum exhibited foetal anaemia (*p* = 0.001). Additionally, all three cases of foetal hydrops were found to have foetal anaemia (*p* = 0.011). Furthermore, four out of the 15 cases with foetal anaemia required blood transfusion.

Paracentesis was performed antenatally for two cases [8, 24], which drained bilious or meconium-stained ascites, and postnatally for two cases [11, 12], which drained bloody ascites.

## Discussion

Finley et al. suggested that a delayed return of foetal bowel from the umbilical cord to the foetal abdomen, potentially resulting in the failure of normal fixation of the small bowel mesentery, could predispose to intrauterine volvulus, as observed in their reported case [15]. Due to the lesser or greater degree of rotation of the gut around the superior mesenteric artery, Cloutier et al. blamed the absence of broad mesenteric attachment [9]. While the majority of postnatal volvulus cases were attributed to malrotation, it is noteworthy that only 17% of intrauterine volvulus cases were associated with malrotation. Additionally, a significant proportion of intrauterine volvulus cases (46%) were found to have no identifiable aetiological or associated anomalies.

Our review identified 22 cases (29%) that were associated with atresia. Notably, the literature cites the rate of atresia in volvulus cases as 25% [5, 38]. However, it remains unclear from the reports whether these atresias occurred secondary to the volvulus or if the volvuli occurred secondary to the atresia. We posit that both scenarios may be true [1, 58].

In the literature, the prenatal ultrasonographic findings of intrauterine volvulus were reported as polyhydramnios, dilated intestinal loops, static abdominal mass, ascites, peritoneal calcifications and increased abdominal circumference [2–4, 17, 26, 30, 32, 36, 38, 40, 41, 46, 51]. In our review, we identified similar lists of prenatal USG findings, although peritoneal calcifications were less frequent while meconium cysts/pseudocysts were more common. Additionally, the literature reported foetal biophysical profile findings as foetal distress with decreased foetal movements and decreased variability during foetal monitorisation, along with foetal cardiac sinus rhythm with anaemia and increased peak systolic velocity in the middle cerebral artery Doppler [2–4, 17, 26, 30, 32, 36, 38, 40, 41, 46, 52] which was mostly observed after 27 weeks of gestation [4, 25, 26, 41]. We found similar foetal biophysical profile findings and more frequent foetal bradycardia, which also increases mortality.

The ultrasonographic signs of intrauterine volvulus can be indicative of several differential diagnoses, including intestinal atresia, meconium ileus, meconium peritonitis, segmental small bowel dilatations, cystic mass lesions such as mesenteric cysts, lymphangioma, or teratoma, Meckel’s diverticulum, duplication cysts, and omphalomesenteric cysts [45, 51, 53]. Definitive diagnosis of volvulus could only be made by the presence of the “whirlpool or snail configuration” or the “coffee bean sign” [1, 3, 4, 26, 32, 36, 38, 40, 43, 49, 53], which are difficult to define [5, 30] and not always associated with volvulus [59]. The sensitivity and specificity of the whirlpool sign were reported as 89% and 92%, respectively in neonatal volvulus cases [54, 60]. We found that the “whirlpool or snail configuration” was reported in 19 cases (24%) and the “coffee bean sign” was reported in 4 cases (5%). Due to the decreased occurrence of specific signs of volvulus and the increased prevalence of nonspecific signs that share many potential differential diagnoses, diagnosing and effectively treating these cases in a timely manner becomes challenging. Consequently, this contributes to elevated mortality and morbidity rates [3–6, 9, 13, 25, 30, 41, 43, 49, 51, 52].

Half of all the cases showed findings of foetal distress. Bartholmot et al. reported that 61.5% of mothers applied with the complaint of decreased foetal movements [1]. The increased risk of foetal loss and foetal distress may occur because of (1) Elevated intraabdominal pressure due to distended bowel and ascites, if present, can impede umbilical venous return and subsequently reduce cardiac output; (2) anaemia resulting from hemoperitoneum can lead to diminished perfusion; (3) the release of toxins from gangrenous or necrotic bowel tissue can exacerbate the physiological stress on the foetus [12, 22]; and (4) fluid escape into the third cavity (dilated bowel lumen) leading to hypovolemic shock and cardiovascular failure [33]. Leung et al. observed that following intrauterine paracentesis of ascites induced by meconium peritonitis, there was an improvement in foetal movements and suboptimal foetal heart rate patterns on non-stress test (NST). This improvement was attributed to the removal of irritant meconium, alleviation of compression on the foetal thorax, and potentially reducing compression on the vena cava, leading to enhanced venous return [24].

The literature mentioned that the outcome of intrauterine volvulus depends on the length of the viable intestine, level of obstruction, presence of meconium peritonitis, associated anomalies, birth weight, and gestational age at birth [17, 30, 38, 49, 54]. We found that the presence of foetal bradycardia at prenatal visits, and presence of meconium cyst or pseudocyst reported in operation were significantly related to decreased survival. Notably, 11 cases (17%) had short bowel syndrome, and two of them died.

One of the factors influencing mortality was the presence of meconium cyst/pseudocyst during operation. In deceased patients, a lower occurrence of dilated bowel in prenatal ultrasonography (44% vs 75%) and a higher incidence of meconium cyst/pseudocyst during operation (36% vs 9%) were observed. Thus, we hypothesize that mortality rises with necrosis and progression of volvulus to perforation, resulting in reduced bowel diameter and formation of meconium cysts.

Foetal anaemia, recognised for increasing mortality and morbidity in both the foetus and newborn, was more prevalent in cases with intrauterine volvulus [13, 36, 38, 42, 43]. While approximately one fourth of intrauterine volvulus cases in the literature were associated with foetal anaemia, we were unable to demonstrate its impact on mortality in our review. However, we observed that hemoperitoneum was a contributing factor to foetal anaemia. In line with the guidance provided by Kornacki et al., we advocate for evaluating the hemodynamic status of the foetus by measuring the peak systolic velocity of the middle cerebral artery in cases presenting with foetal bowel pathology, particularly in foetuses with intrauterine volvulus [36].

In severe cases, volvulus can manifest early in pregnancy, leading to the development of intestinal atresia and meconium peritonitis. Such instances may necessitate immediate intervention or preterm delivery due to the presence of foetal distress [6, 18]. Additionally, severe intrauterine volvulus led to ischaemic necrosis of intestines and might activate both feto-placental and hypothalamic release of stress hormones that results in uterine contractions and preterm labour [3, 20, 28, 30, 32, 42, 55]. We found that in the deceased patients, the preterm labour was more frequent (54% vs 21%), while urgent caesarean section and preterm newborn ratio was less frequent (27% vs 47%, 58% vs 78%, respectively). Therefore, we deduced that in cases where survival was achieved, obstetricians closely monitored these patients to detect signs of foetal distress and promptly conducted urgent caesarean sections to prevent foetal demise.

Two studies highlight that delays in diagnosing and treating intrauterine volvulus can lead to high complication rates, including loss of intestinal segments and the development of short bowel syndrome [17, 18]. Some authors suggest that delivery can be promptly performed via caesarean section after 34 weeks of gestation, while the timing of delivery before 34 weeks depends on the severity of the foetal condition and the need for prophylactic measures for foetal lung maturation [43, 45], Additionally, in cases where prenatal diagnosis is established, some authors recommend preterm delivery and emergent surgical intervention [26, 42]. However, others emphasize that the timing of delivery should be based on obstetrical indications, given the uncertainty in differentiating between intestinal atresia, meconium ileus, and volvulus [14, 17]. It’s also suggested to closely monitor foetuses presenting with signs of bowel obstruction and to consider urgent caesarean section in the presence of acute abdominal pathology or non-reassuring foetal status [1, 21, 55] when findings of acute abdominal pathology such as the heterogeneous echogenicity within the dilated bowel or the disappearance of peristalsis are presented [21] or when there are findings of non-reassuring foetal status [52, 55]. For instance, Herrera et al. recommend urgent delivery in the presence of specific indicators such as ascites, absence of intestinal peristalsis, decreased foetal movements, or sudden changes in bowel diameter [51], while Alvarez et al. suggest delivery when findings of thoracic compression are detected [14]. We recommend closely monitoring foetuses exhibiting signs of intestinal obstruction and administering betamethasone therapy for pulmonary maturity, particularly given the observed frequency of preterm labour. Urgent caesarean section should be considered as an option before foetal biophysical profile findings deteriorate further. It’s important to note that planned preterm delivery solely based on ultrasound findings is not advised, as these findings may also be indicative of conditions such as atresia or meconium peritonitis that do not necessitate urgent intervention. However, the likelihood of preterm delivery may still be higher due to a greater incidence of non-reassuring foetal tests.

However, our study has several limitations. Firstly, we were unable to access all the available case reports and case series, and our investigation was confined to English-language literature. Secondly, our data were constrained by what was reported, resulting in missing information on many features and findings. Thirdly, we assumed that certain non-reported features and findings were normal, such as the absence of necrotic bowel or mesenteric anomalies. If authors did not explicitly mention necrosis of bowel segments, we presumed the bowel to be viable. At the same time, it is possible that mesenteric anomalies were not universally investigated or documented by all surgeons, leading to potential oversight of many cases.

Due to the predominance of non-specific signs over specific signs of volvulus, which can mimic various differential diagnoses, diagnosing and treating these cases in a timely manner posed significant challenges. We concluded that the severity of volvulus may contribute to preterm labour, thereby increasing mortality. We posit that mortality escalates with necrosis and progression of volvulus to perforation, resulting in a reduction in bowel diameter and formation of meconium cysts. Consequently, we recommend close monitoring of foetuses exhibiting signs of intestinal obstruction, administration of betamethasone therapy for pulmonary maturity given the frequent occurrence of preterm labour, and consideration of urgent caesarean section before foetal biophysical profile findings deteriorate further.

## Supplementary information

Data set
