# Supplementary material for: Intrauterine volvulus: systemic review of the literature with pooled analysis
Source: J Perinatol. 2024 May 4;44(11):1543–51. doi: 10.1038/s41372-024-01984-6 (PMC11519003; doi:10.1038/s41372-024-01984-6)
Supplement: Supplementary file 1 — Data set [file 41372_2024_1984_MOESM1_ESM.docx]

**Dataset (File S1):** List of cases from a review of the literature (57 case reposts/case series, a total of 88 cases).

| No. | Author (year) | Prenatal USG time and findings | Fetal biophysical findings | GAB | BW | G | PE | Radiology | Op. findings | Cause: | Op. | Ass. | O |
| --- | --- | --- | --- | --- | --- | --- | --- | --- | --- | --- | --- | --- | --- |
| 1 | Seward (1978) [7] |  | Fetal bradycardia | term | 2700 g | M | AD, FH, Cy,  Lethargic, Multiple petechiae, Pupils mid-position & non-reactive, extremities flaccid, seizures | X-ray: GA except stomach | Volvulus, NB,  large amount HP | - | A | FH, Int. & Vent., FA (Hb:7.1 g/dl, Hct:22%); BT | B |
| 2 | Baxi (1983) [8] | 25 wk: N,  34 wk: minimal PHA,  Repeated: moderate PHA, AD, Asc.,  Intrauterine growth retardation,  Cystic loculation in right hypochondrium,  Liver and lungs were compressed,  36 hours later: increased PHA, AD, Asc.  Disappearance of previous cystic mass (perforation),  Further compressed fetal thorax |  | 34 wk 2 d | 2200 g | F | AD |  | PC (filled with meconium and necrotic debris),  Perforation at 55 cm segment of jejunum, remaining 35 cm ileum with impacted meconium, microcolon | MI | A | Antenatal paracentesis (MSAF) | G |
| 3 | Cloutier (1983) [9] | 35 wk: Large AM (10.5 cm), (cystic, with septations and a fluid-debris level) | 33 wk: treated PL,  35 wk: PROM | 35 wk | 2500 g | F | AD, CS, AM (7 cm) | X-ray: a large AM with long AFL | PC (A large cystic mass filled with green fluid),  NB from mid-duodenum to mid-transvers colon. | - |  | Meconium aspiration that needs resuscitation, | B |
| 4 | Samuel (1984) [10] | 33 wk: PHA, AM, DB  One dilated loop of bowel entering the mass | N | 34 wk |  |  | AD, AM |  | Volvulus (resected)  Peritonitis | - | E |  | G |
| 5 | Nogami (1985) [11] |  |  | term | 3230 g |  | AD, FH | X-ray: GA without calcification, | Volvulus, NB from the distal duodenum to the distal ileum  (Postmortem) | - |  | Complicated vaginal delivery (shoulder dystocia and tearing of umbilical cord),  Int. & Vent.,  FA (Hct:20%),  Paracentesis: 55 mL grossly bloody fluid. | B |
| 6 | Witter (1986) [12] | 34 wk: LFM | 34 wk: DFM,  Positive oxytocin challenge test,  NRP without tachycardia with DV. | 34 wk | 2200 g | F | TA, Asc., CS,  edema of abdominal and chest wall but sparing of the face and extremities, | X-ray: Chest: bilateral plevral effusion,  Contrast enema: High cecum,  Narrow colon,  Upper GIS series: obstruction in the mid-jejunum. | Volvulus  (59 cm of distal jejunum and 12 cm of proksimal ileum - resected) | - | A | At birth: color remained dusky, poor respiratory efforts, heart rate fell rapidly,  Int. & Vent. Acidosis,  Paracentesis: 190 ml old dark blood (HP).  FA (Hct 20%); BT,  Bilateral chest tubes, | G |
| 7 | Ho (1990) [6] | DB, Asc. | DFM | 35 wk | 2398 g | M | AD, CS |  |  |  | A | HP, FA, RD | G |
| 8 | Suzumori (1990) [6] | PHA, AM | DV on CTG | 36 wk | 2820 g | M | AD |  |  |  | A | RD | G |
| 9 | Usmani (1991) – 1 [13] | 28 wk: PHA | PL | 29 wk | 1200 g | F | AD, TA, BGA | X-ray: air-filled loops of bowel without distention, 36 hrs: single, isolated DB (massive)  Contrast enema: N colon (cecum at right upper quadrant) | Volvulus, NB | - | A |  | G |
| 10 | Usmani (1991) – 2 [13] |  | DFM, Low biophysical profile score | 34 wk | 1800 g | F | AD, TA, CS, BGA | X-Ray: a few air-filled loops at the left of the abdomen  USG: N orientation of mesenteric vessels. | Volvulus  NB (from 25 cm below ligament of Treitz to within 1 cm of the ileocecal valve) | - | E (Hartmann pouch) | Third of triplets, MSAF,  At birth: Bradycardic, pale and hypotensive, Int. & Vent.,  FA (Hct 32%); BT | G |
| 11 | Alvarez (1991) [14] | 30 wk: Asymmetric growth retardation, AM (5.2 cm), Oligohydramniosis,  34 wk: AM enlarged (7.1x6.0x7.3 cm), elevation of diaphragm, compression of intrathoracic structures. |  | 34 wk | 1400 | F | AD, BGA (positive for bilirubin and blood), failure to pass meconium | X-ray: Diffuse haziness with a large non-calcified radiolucent density in the left hypochondrium extending to midline.  USG: A large septated AM contsisting of cystic and solid elements that extend from abdomen to pelvis | Large PC (75 mL of green liquefied meconium), a twisted loop of the proximal ileum at base of the cyst wall, at least 2 sites of perforation, a segment of intestine was adherent to the cyst. Remaining: 27 cm of small bowel. | - | A | Twin, SBS | G |
| 12 | Finley (1992) [15] | 13 wk: Umbilical herniation,  15 wk: N umbilical cord, N fetus,  20 wk: DB, Exaggerated peristalsis |  | 36 wk | 2835 g | M | AD, BGA |  | Volvulus,  NB (Only 15 cm intact small bowel),  Absent cecum and ileocecal valve (type 3),  microcolon | Atr. | A | Antenatal: Late return of fetal bowel from umbilical cord to fetal abdomen  Postoperative: SBS | ? |
| 13 | Dell’agnola (1993) -1 [16] | AM, DB |  |  |  |  |  |  | Volvulus |  |  |  |  |
| 14 | Dell’agnola (1993) – 2 [16] | PHA, DB, Asc. |  |  |  |  |  |  | Volvulus  MP |  |  |  |  |
| 15 | Dell’agnola (1993) – 3 [16] | AM, DB, Asc., |  |  |  |  |  |  | Volvulus  perforation |  |  |  |  |
| 16 | Dell’agnola (1993) – 4 [16] | Asc. |  |  |  |  |  |  | Volvulus  Peritonitis | Atr. |  |  |  |
| 17 | Mercado (1993) [17] | 22 wk: N  27 wk: PC (fluid and debris filled)  ? wk: DB, mild PHA, discrete cystic mass containing echogenic material (5 cm),  34 wk: the walls of the cystic structure became echogenic and several peritoneal calcifications |  | 38 wk |  |  | AD | X-ray: Multiple DB displaced by AM  Contrast enema: Microcolon, small amount of contrast refluxed into terminal ileum | Thin mesenteric band between mesentery and umbilicus, atretic bowel loops volvulized around band. MC (4.5 x 4 x 3.5 cm) surrounded by loops of atretic distal jejunum and proximal ileum. Adhesions. Only 60 cm viable small bowel remained. | Atr.  Band | ? | Twin pregnancy,  SBS | G |
| 18 | Black (1994) [18] | DB with thickened walls and were aperistaltic | PL | 34 wk | 2170 g | M |  | X-ray: GA except DB (proksimal bowel) | DB, volvulus (foreshortened mesentery), NB | MA | A | Follow-up: Adhesive bowel obstruction that required lysis of adhesions. | G |
| 19 | Bronshtein (1996) – 1 [19] | 16 wk: marked DB with anechoic content | LFM |  |  |  |  |  | Postmortem: Volvulus with a large blood clot |  |  | Ambigous genitalia,  46XY, Anal atresia, | PT |
| 20 | Bronshtein (1996) - 2 [19] | 16 wk: Hydrocephalus, DB, Small for gestational age, |  |  |  |  |  |  | Postmortem: Volvulus |  |  | Anal atresia | PT |
| 21 | De Felice (1997) [20] | 27 wk: DB | PL | 27 wk | 970 g | F | 24 hrs: AD, BGA, failure to pass meconium | X-ray: dilated stomach and moderate DB (proximal bowel) with AFL | Volvulus, NB | - | A |  | G |
| 22 | Miyakoshi (1998) [21] | 34 wk: DB (hypoechogenic, diameter of 21mm),  35 wk: DB with intraluminal sludge and disappearance of peristaltic movements,  Mild Asc. | 35 wk: LFM,  NRP with DV on CTG | 35 wk | 2128 g | M | AD, TA,  Rebound and tenderness | X-ray: findings of small bowel obstruction | DB, mild Asc.,  DB twisted around proximal to atresia at the distal segment of ileum (strangulated) | Atr. | A |  | G |
| 23 | Yoo (1999) – 1 [4] | 35 wk: WS, markedly DB  presented as large oval cystic, AM, No flow signal within or between loops (Doppler USG) |  | 35 wk 4 d | 2810 g | F | AD (moderate),  CS |  | Volvulus, NB, Meconium stained Asc., | M | A,  LP |  | G |
| 24 | Yoo (1999) – 2 [4] | 30 wk: AM, Mildly DB, WS Cystic expanded bowel,  Mild PHA  35 wk: Cystic AM, worsened DB (proximal bowel), increased PHA |  | 37 wk 1 d | 2480 g | M | AD | USG: DB, Focal cystic distension | Non- gangrenous volvulus involving middle segment of small intestines | M,  Atr. | A (Atr)  LP |  | G |
| 25 | Crisera (1999) [2] | N | N | 37 wk |  | F | AD, TA, CS, BGA, bloody stool | X-ray: Paucity of distal bowel gas | Volvulus, NB,  After resection, 16 cm jejunum and 17 cm distal ileum remained. | M | E |  | G |
| 26 | Morikawa (1999) [6] | 31 wk: DB | DFM, DV on CTG | 31 wk 6 d | 1774 g | F | AD, bloody stool | USG: DB filled with fluid. | Volvulus (32 cm distal to Treitz – 8 cm prox to ileocecal valv) | MA | A | FA, RD  One umbilikal artery | G |
| 27 | Ogunyemi (2000) [22] | 36 wk: PHA, DB, AD  dilated stomach,  dilated double loop of bowel | DV, FD on CTG. | 36 wk | 2085 g | M |  |  | Volvulus, NB, HP | M, MA,  Atr. | E | Twin pregnancy, Bilious stained amniotic fluid,  SBS (Gastrostomy + TPN) | G |
| 28 | Kubota (2000) [23] | 37 wk: single anechoic bubble in the fetal abdomen |  | 38 wk | 2640 g | F | A gradual increase in AD | Scopy (?): single bubble-such as DB that showed peristaltic movement and was filled with swallowed air,  Contrast studies: (upper and lower GIS) no dilatations. | Volvulus (massive accumulation of meconium and swallowed air but not strangulated),  Atresia 50 cm from terminal ileum. | Atr. | A |  | G |
| 29 | Leung (2001) [24] | 20 wk: N  33 wk: AD, MP (loculated Asc. with intraperitoneal debris)  34 wk: AD, MP (7.8x7.3 cm) (loculated collection of Asc. including debris in the left abdominal cavity)  No stomach bubble,  Elevated diaphragm | 33 wk: N  34 wk: DFM,  FD on CTG  PL (just after the intervention) | 34 wk | 3000 g | M |  |  | Volvulus with perforation, 3 cm proksimal to ileocecal valve | - | E | Twin pregnancy,  Intrauterine paracentesis of Asc. (MP),  Int. & Vent.  Follow-up: E closure (1 month later) | G |
| 30 | Uerpairojkit (2001) [25] | 33 wk: AD, Fixed DB, AM, PC | DFM, DV and NRP on CTG, PL | 33 wk | 2215 g | M | AD, Rapid fall in heart rate | X-ray: GA | Volvulus, NB, Meckel divertikulum | - | A | RD | G |
| 31 | Has (2002) [26] | 34 wk: PHA, Mild Asc.,  a long segment of DB forming WS  Doopler: no identifiable flow signal within and between DB  34+5 wk: decreased volume of DB loops, WS had disappeared,  Asc. of heterogeneous echogenicity (MP). | PL | 34 wk 5 d | 2000 g | M | AD, CS |  | Volvulus, perforation,  30 cm of bowel resected | Atr. | E | Intestinal duplication | B |
| 32 | Jequier (2003) [27] | 27 wk: DB, AM (complex, subumbilikal) (5.5 cm),  29 wk: AM became cystic,  Increased DB, PHA |  | 33 wk |  |  |  | X-Ray: AD, AM with peritoneal calcifications. | Volvulus, NB, perforation with MP | - |  |  | G |
| 33 | Allahdin (2004) [28] | N | 39 wk: N labor;  no fetal heart beat | 39 wk | 3480 g | F | No signs of abdominal pathology |  | Volvulus, NB | - |  |  | ID |
| 34 | Trachsel (2007) [29] |  |  | 38 wk |  |  |  |  | Volvulus, NB | M |  | CF, Focal biliary cirrhosis, Intestinal calcifications | ID |
| 35 | Molvarec (2007) – 1 [30] | 32 wk: slightly DB (max. 15 mm), Mild PHA,  36 wk: Asc., Markedly increased DB (45 mm), AD and amniotic volume, |  | 36 wk | 2150 g | F | AD, TA, | X-ray and USG: findings of intestinal obstruction and perforation | Volvulus, NB, MP perforation of small bowel at 80 cm | M | A | SBS | G |
| 36 | Molvarec (2007) – 2 [30] | 36 wk: Several DB loops (max. 20 mm)  Daily Follow: DB (max. 25mm), markedly increased AD, peristalsis. | Reactive CTG | 36 wk 3 d | 2350 g | M | AD, TA, BGA | USG: DB (max. 27 mm), | Volvulus, NB (20 cm) | M | A |  | G |
| 37 | Rajab (2007) [31] | 33 wk: Dilated stomach, slightly DB, | DFM, PROM, FD on CTG | 34 wk | 2200 g | M | AD, TA, BGA | USG: stomach is distended, DB (proximal bowel), spiral appearance (WS), small amount of Asc., | Volvulus, HP, NB (22 cm) | - | A |  | G |
| 38 | Park (2008) [32] | 33 wk: DB with CBS. | PL | 33 wk 2 d | 2690 g | M | AD, CS, BGA | X-ray: GA except in stomach.  CT: Marked DB, large amount of HP | Volvulus, NB, (40 cm distal to ligament Treitz, 15 cm proximal to ileocecal valve)  Narrow, meconium filled distal ileum | MI | A,  Saline irrigation for MI | Postoperative gastrografin enema for MI | G |
| 39 | Steffensen (2008) [33] | 15 wk: fetal Asc.  25 wk: increased fetal Asc., DB, bilateral club foot,  38 wk: fetal demise | DFM,  Fetal demise | 38 wk | 2780 g | M | AD |  | Volvulus, Severely DB loops, Red-brown fluid in DB segments,  Atresia (45 cm from treitz) | M,  Atr.  MA |  | Fetal arthrogryphosis | ID |
| 40 | Noreldeen (2008) [34] | 20 wk: N,  31 wk: AD, PHA, Fetal Asc.,  DB loops  Doppler: umbilical artery: positive end-diastolic flow. | DFM (31 wk)  DV on CTG,  USG: LFM, increased PSV in MCA (FA) | 31 wk | 1860 g | M | AD, CS | USG (2 h): large amount of free fluid mixed with echogenic particles, | Volvulus, NB, HP | - | A | FA, Int. & Vent. | G |
| 41 | Durand (2008) -1 [35] | 34 wk: PHA, a long segment of DB, WS, Doppler: no flow sign within or between the dilated loops. | NRP on CTG Emergent cesarean section | 34 wk | 2180 | F | AD | X-ray: GA below stomach | Midgut volvulus, intestinal perforation, resection of gangrenous segment | MI | E | CF, Sepsis, seizures, cerebral palsy with hemiplagia, MR, cortical blindness, hepatic cirrhosis, recurrent pulmonary infections (death at 10 y) | G |
| 42 | Durand (2008) - 2 [35] | 39 wk: a long segment of DB, WS, Doppler: no flow sign within or between the dilated loops. | NRP on CTG Emergent cesarean section | 39 wk | 2660 | F | AD | X- ray: GA below stomach | Midgut volvulus, resection of gangrenous segment | MI | E | CF, intestinal obstruction after stoma closure necessitated bowel resection. | G |
| 43 | Durand (2008) – 3 [35] | 34 wk: a long segment of DB, WS, Doppler: no flow sign within or between the dilated loops. | planned cesarean section | 34 wk | 2190 | F | AD | X- ray: GA below stomach | Midgut volvulus, resection of gangrenous segment | MI | E | CF, intestinal obstruction after stoma closure necessitated stoma. | G |
| 44 | Kornacki (2010) [36] | 32 wk: FH, PHA,  Mild hydrothorax,  Thick Asc. around liver,  Hyperechogenic, longitudinal structure with thick walls (DB?) | 32 wk: Increased PSV in MCA | 34 wk | 3010 g | F | AD | USG: AM (4.4 x 6.5 cm), | Volvulus,  Significantly DB loop filled with meconium (25 cm),  Hypoplastic distal small bowel and colon,  Adhesion between ileum and liver | - | A | FH,  33 wk: FA (Cordocentesis: Hct 24%, postnatal Hb:9.9 g/dl, Hct:32%); BT  Plevral effusion,  Re-laparotomy (7 days later): biliary peritonitis, no source was found.  39^th^ day: sepsis | G |
| 45 | Valladares (2010) [37] | 32 wk: AM (at left hemiabdomen) with mixed echogenicity (MC), abnormal umbilical artery,  Fetal MRG: 72x58mm, heterogenous, mesenteric AM without necrosis, distortion of small intestines to the left. | PL (32 wk) | 32 wk 6 d | 1980 g |  |  |  | Volvulus, Perf., secondary meconium PC, NB (10 cm) at 10 cm from ileocecal valve. | - | A | Twin, SBS,  Postoperative 3^rd^ day: Re-operation and segmental resection, anastomosis;  51^st^ day: resection (5 cm bowel including ileocecal valve) re-anastomosis because of stenosis,ileostomy and colostomy,  Death at 7^th^ month | B |
| 46 | Yu (2013) [38] | 37 wk: DB (long segment) forming WS,  Doopler USG: Mesenteric vessels could not be seen within twisted loops  Large oval cystic AM (8x5x4 cm),  Fetal Asc., Mild PHA |  | 37 wk | 2900 g | F | AD, TA, CS | USG: AM in left upper quadrant, DB, Asc., Convoluted bowel | Volvulus, NB, Asc. (200 ml) | Atr. | A | FA, SBS (postoperative) | G |
| 47 | Shankar (2013) [39] | 33wk: DB with multiple fluid levels |  | 33-34 wk | 1800 g |  | Mild AD (soft, non-tender),  BGA | X-ray: Findings of small bowel obstruction | Volvulus, NB (15 cm) | - | A | Re-operation because of Anatomosis leakage | G |
| 48 | Ohuoba (2013) [40] | 24 wk: N  36 wk: Fetal mega-cisterna magna 14-16 mm,  DB at lower digestive tract. | DFM, DV, FD on CTG | 37 wk 6 d | 3353 g | M | AD, TA, dark bloody gastric aspirate | X-ray: Asc., GA except small stomach,  USG: multiple DB, filled with fluid,  Upper GIS series: malrotation. | Volvulus, Markedly DB (ischemic jejunum), at distal end of atresia (type 3A) 57 cm intussusception, 17 cm jejunum and 50 cm ileum was patent. | M,  Atr. | A | FA, MSAF, Apnea and desaturation periods (intubated), Hypoglisemia, Leukocytosis | G |
| 49 | Pederiva (2013) [5] | DB  33 wk: Moderate Asc. | Fetal bradycardia on CTG,  Increased PSV in MCA (FA) | 33 wk | 2070 g | M | AD, CS, bloody gastric aspirate | X-ray: GA except stomach  USG: WS, large amount of free fluid mixed with echogenic particles | Volvulus (15 cm) distal to Type I atresia of prox. Ileum, 2^nd^ atresia at the base of volvulus,  3 type I atresia  Perf. | Atr. (Primary or secondary?) | A (Atr)  E | FA | G |
| 50 | Davidson (2013) [41] | 26 wk: N | FD on CTG, PL | 32 wk 3 d | 2000 g | F | AD, CS |  | Postmortem: Volvulus (42 cm) | - |  | FA, Int. & Vent. (Cyanotic),  Recurrent pulmonary hemorrhages | B |
| 51 | Chung (2013) [42] | 31 wk: N,  37 wk: AD, DB (max. 25 mm), Asc., Peritoneal calcifications, | 31 wk: Placenta Previa  37 wk: DFM, bloody vaginal discharge,  DV with late decelerations on CTG | 37 wk | 2630 g | M | AD, CS,  Tachycardia  Tachypnea, | X-ray and USG: MP, volvulus | Volvulus, NB, (70 cm distal to Treitz ligament, 5 cm proximal to ileocecal valve), HP, meconium stained Asc.  Biopsies from colon | - | E | FA, Maternal gestational thrombocytopenia, Signs of ganglion cell immaturity at descending and sigmoid colons  (42^nd^ day: E closure) | G |
| 52 | Nakagawa (2014) [43] | 34 wk: FA, LFM | DFM, NRP on CTG, Increased PSV in MCA (FA) | 34 wk 4 d | 2494 g | F | AD, CS | X-ray: GA, AD  Contrast enema: suspected stenosis of terminal ileum | Volvulus, NB, HP, resection of 40 cm jejunum, 2 cm distal ileum remained. | - | A | Severe FA | G |
| 53 | Artul (2013) [44] |  | PL | 35 wk | 2666 g | F | AD, Grunting | X-ray: AD, GA except stomach,  Upper GIS study: barium stopped at the level of right lower quadrant, ‘bird beak’ sign with filling defect, around structure around this end loop,  USG: a target lesion consistent of 2 loops of bowel, pneumotosis of the bowel wall, twisting of the vessels | Volvulus, Perf. | - | E |  | G |
| 54 | Kaba (2015) [3] | 30 wk: AM (lower abdomen) (55x50 mm) | Severe sinus rhythm on CTG, increased PSV in MCA (FA),  PL | 30 wk | 1625 g | F | AD | X-ray: AD, DB on the left side.  USG: AM (60 mm) | Volvulus,  Twisted bowel loop became a necrotic cystic mass, 20 cm proximal to ileocecal valve | - | A | FA, Int. & Vent. | B |
| 55 | Sciarrone (2016) – 1 [45] | 20 wk: PHA, DB, AD |  | 35 wk | 2410 g | F |  |  | Volvulus (30 cm from Treitz), peritonitis, multiple ileal atresias, PC | Atr | E | Peritonitis, enterostomy closure at 1 month. | G |
| 56 | Sciarrone (2016) – 2 [45] | 24 wk: DB,  27 wk: probable perforation of the DB |  | 39 wk | 3370 g | F |  |  | Volvulus, ileal atr (80 cm from Treitz), Meckel diverticulum | Atr | A |  | G |
| 57 | Sciarrone (2016) -3 [45] | 23 wk: DB, Asc, AD  30 wk: amniotic fluid rapidly increased, peristalsis disappeared, Asc appeared. |  | 30 wk | 1630 g | F |  |  | Volvulus, ileal atr (50 cm from Treitz), MP | Atr, MI | A | CF (respiratory complications), Peritonitis, single umbilical artery | G |
| 58 | Sciarrone (2016) -4 [45] | 33 wk: WS, Gastric dilatation, DB, AD |  | 33 wk | 2300 g | M |  |  | Volvulus | MI | A | CF, dilated cistern magna, hyperechogenic spots | G |
| 59 | Sciarrone (2016) -5 [45] | 32 wk: PHA, CBS, Gastric dilatation, DB, AD, PC |  | 34 wk | 3150 g | M |  |  | Volvulus (40 cm from Treitz) (3 complete twist), MP | MI | E | CF(respiratory complications), Peritonitis, enterostomy closure at 1 month | G |
| 60 | Sciarrone (2016) -6 [45] | 32 wk: CBS, DB, PC |  | 32 wk | 2190 g | M |  |  | Volvulus (25 cm from Treitz) (2 complete twist) forming PC | - | A | Peritonitis | G |
| 61 | Sciarrone (2016) -7 [45] | 30 wk: PHA, DB, |  | 31 wk | 1650 g | M |  |  | Volvulus (60 cm from Treitz) (3 complete twist), MP, DB and perf. | - | A | 3 wk later peritonitis and NB, massive ileal and colonic resection, gastrostomy. | G |
| 62 | Sciarrone (2016) -8 [45] | 32 wk: DB, Asc, AD |  | 32 wk | 1930 g | M |  |  | Volvulus, ileal atr | Atr | A | Peritonitis, intestinal obstruction and ileal resection at 1 month | G |
| 63 | Best (2018) [46] | 31 wk: moderately dilated fetal stomach, DB, aperistalsis, Doppler: N, Daily USG: DB up to 18 mm with increasing echogenicity, min peristlasis, loss of definition in the bowel wall, indicating edema, WS, CBS | 31 wk 5 d: DFM, NRP on CTG, mild fetal tachycardia,  32 wk: PL | 32 wk 2 d | 2450 g | F | N | Upper GIS contrast study: no malrotation.  X-ray (day 2): pneumoperitonium | Volvulus, MP, NB | - | A |  | G |
| 64 | Bartholmot (2018) – 1 [1] | 27 wk: WS, fluid-meconium level, DB, MP(calcifications, Asc, PC) |  |  |  |  |  |  | Volvulus | MI |  | CF | PT |
| 65 | Bartholmot (2018) – 2 [1] | 26 wk: WS, DB, MP (calcifications, Asc, PC) | DFM | 37 wk |  |  |  |  | Volvulus, Atr. | Atr |  |  | G |
| 66 | Bartholmot (2018) – 3 [1] | 34 wk: WS, fluid-meconium level, DB, MP (calcifications, Asc, PC) | DFM, NRP | 35 wk |  |  |  |  | Volvulus, Atr. | Atr |  |  | B |
| 67 | Bartholmot (2018) – 4 [1] | 27 wk: WS | DFM |  |  |  |  |  | Volvulus | MI |  | CF | PT |
| 68 | Bartholmot (2018) – 5 [1] | 28 wk: WS, fluid-meconium level, DB, MP (calcifications, Asc, PC) | DFM | 38 wk |  |  |  |  | Volvulus, Atr. | Atr |  |  | G |
| 69 | Bartholmot (2018) – 6 [1] | 32 wk: DB, MP (calcifications, Asc, PC) | DFM | 33 wk |  |  |  |  | Volvulus | - |  | Twin | G |
| 70 | Bartholmot (2018) – 8 [1] | 33 wk: fluid-meconium level, DB, MP (calcifications, Asc, PC) |  | 35 wk |  |  |  |  | Volvulus, Atr. | Atr |  |  | G |
| 71 | Bartholmot (2018) - 10 [1] | 31 wk: WS | DFM | 38 wk |  |  |  |  | Volvulus | - |  |  | G |
| 72 | Bartholmot (2018) -11 [1] | 31 wk: WS, DB, MP (calcifications, Asc, PC) | DFM, NRP | 34 wk |  |  |  |  | Volvulus, Atr. | Atr |  |  | G |
| 73 | Bartholmot (2018) -12 [1] | 22 wk: WS, MP (calcifications, Asc, PC) |  | 36 wk |  |  |  |  | Volvulus, Atr. | Atr |  |  | G |
| 74 | Bharti (2018) [47] |  | LFM (1 month earlier), PL, PROM, DV | 36 wk |  | F | AD, CS, lethargic, mild hypotonia, pale, petechial over eyelids |  | Volvulus, NB | - | A, G | DIC, NB,  thrombocytophy, Vitreous hemorrhages, SBS, | G |
| 75 | Gultekin (2019) [48] | 12 wk; Mesenteric Cyst |  | term |  |  | BGA, CS | CT: Mesenteric cyst | Ileal torsion, NB | M | E | NB, 15 days later: anastamosis | G |
| 76 | Vazquez (2020) [49] | N | DFM, DV | 33 wk | 2128 g | F |  | X-ray: paucity of air at right and DB at left  USG: DB, mild bowel wall edema  Doppler USG: absence of flow in affected bowel loops, abnormal SMA and SMV relation | Volvulus of 3 loops, ladd bands, NB | M, MA | A, LP | RD, Int. & Vent., metabolic acidosis, NB | G |
| 77 | Park (2020) [50] | 24 wk; DB, Asc. | FD, deceleration on CTG | 34 wk | 2850 g | M | AD | X-ray: GA | Volvulus, MP, DB, Perf. | - | A | RD, Int. & Vent.,  15 days later: adhesiolysis, ileostomy.  Cholestasis (TPN related), difficulty in extubation | G |
| 78 | Herrera (2020) [51] | 26 wk; DB (lower GIS), WS | PL (22 wk; prevented), PROM | 29 wk | 1458 g | M | N |  | Volvulus, NB, Perf. | M | E | TRAP treatment | G |
| 79 | Nagano (2021) [52] | 35 wk; DB, LFM, DGB, WS, CBS | DFM, DV | 35 wk | 2346 g | F | AD | X-ray: GA (except gastric bubble)  Upper GIS contrast study: normal position of Treitz lig.  Contrast enema: microcolon-like appearance (appendix in right lower abdomen) | Volvulus, DB, yellowish, cloudy asc., NB (50 cm ileum), | - | A | Respiratory acidosis, Int. & Vent. | G |
| 80 | Gercel (2021) [53] | 24 wk; DB |  | 38 wk | 3600 g | M | BGA, AD (min.) | X-ray: AFL | Volvulus (bowel perfusion was normal) | M | LP |  | G |
| 81 | Khoury (2021) [54] |  |  | Term | 2700 g | M | AD | X-ray: AFL, DB  USG: DB with hyperactivity movement | Volvulus (ileum), perf. (volvulated segment), meconium peritonitis | - | E | Respiratory effort worsened with increasing abd. diameter | G |
| 82 | Mise (2022) – 1 [55] | 30 wk; DB, Asc. (presented later, when dilated bowel shrunk) | DFM, PL, DV, increased MCA PSV, FA | 31 wk | 1626 g | M |  |  | Volvulus (widespread NB), Bloody Asc. | - | E | Remaining small intestines 45 cm | G |
| 83 | Mise (2022) – 2 [55] | 34 wk; DB (upper GIS), Asc. |  | 34 wk | 2830 g | F |  |  | Volvulus, Asc., NB, Perf. | - | E | Remaining small intestines 85 cm | G |
| 84 | Sahin-Uysal (2022) [56] | 24 wk: focal DB with an increased wall echogenity  27 wk: DB progressed,  28 wk 5 d: DB progressed (max. 30 mm), PHA | 28 wk 5 d: DFM,  32 wk: PL | 32 wk | 2320 g | F | AD, BGA, CS, | X-ray: AFL,  USG: fluid distention at stomach and intestines, DB without abscess | MC (cystic, stiff, yellow-colored, fibrinous pouch), unused colon, distal jejunum and ileum was absent, only 25 cm jejunum left, jejunal and ileal ends were atretic | - | E | SBS, Sepsis (35^th^ day) | B |
| 85 | Ashworth (1988) - 1 |  |  | 20 wk |  | F |  |  | (necropsy) volvulus (lower ileum) | - |  |  | Mis |
| 86 | Ashworth (1988) - 2 |  | Fetal heart was recorded as disappearing | 22 wk |  | M |  |  | (necropsy) volvulus (complicated; across two loops of bowel) | - |  |  | Mis |
| 87 | Ashworth (1988) - 3 |  |  | 22 wk |  | M |  |  | (necropsy) volvulus (midileal), Subcapsular hematoma, bleeding into abdominal cavity | - |  |  | Mis |
| 88 | Ashworth (1988) - 4 |  |  | 22 wk |  | M |  |  | (necropsy) volvulus (lower ileum) (complicated) | - |  |  | Mis |

Abbreviations at Headings:

USG: Ultrasonography, GAB: Gestational age at birth, BW: Birth weight, G: Gender, PE: Physical examination, Op: Operation, Ass.: Associated findings and features, O: Outcome.

Abbreviations at Column “Prenatal USG time and findings”:

PHA: polyhydramniosis, AD: Abdominal distention (for antenatal USG circumference of abdomen >95 percentile), Asc.: Ascites, AM: Abdominal mass, DB: Dilated bowel, LFM: Loss of fetal movements, PC: Pseudocyst, WS: Whirlpool sign, MP: Meconium peritonitis, FH: Fetal Hydrops, FA: Fetal Anemia, DGB: Dilated gastric bubble, CBS: Coffee-bean sign.

Abbreviations at Column “Fetal biophysical findings”:

PL: Preterm Labor, PROM: Premature rupture of membranes, DFM: decreased fetal movements, NRP: Non-reactive pattern, DV: Decreased or no variability of fetal heart rate, CTG: Cardiotopography/Non-stress test, LFM: Loss of fetal movements, FD: Fetal Distress, PSV: peak systolic velocity, MCA: middle cerebral artery, FA: Fetal Anemia.

Abbreviations at Column “Gender” (G):

F: female, M: male.

Abbreviations at Column “Physical examination” (PE):

AD: Abdominal distention, FH: Fetal Hydrops, Cy: cyanotic, CS: Cullen”s sign, AM: Abdominal mass, TA: Tense abdomen, Asc.: Ascites, BGA: Bilious gastric aspirate.

Abbreviations at Column “Radiology”:

GA: Gasless abdomen, AM: Abdominal mass, AFL: Air-fluid level, DB: Dilated bowel, GIS: Gastrointestinal System, AD: Abdominal distention, USG: Ultrasonography, WS: Whirlpool sign, Asc.: Ascites, CT: computerized tomography, HP: Hemoperitonium, MP: Meconium peritonitis, SMA: Superior mesenteric artery, SMV: Superior mesenteric vein, lig.: ligament.

Abbreviations at Column “Operative findings”:

NB: Necrotic bowel, HP: Hemoperitonium, PC: Pseudocyst, MP: Meconium peritonitis, MC: Meconium cyst, DB: Dilated bowel, Asc.: Ascites, atr: Atresia, perf.: perforation.

Abbreviations at Column “Cause”:

MI: Meconium Ileus, Atr: Atresia, MA: Mesenteric anomaly, M: Malrotation.

Abbreviations at Column “Operation” (Op.):

A: Anastomosis, E: Enterostomy, Atr: Atresia, LP: Ladd procedure, MI: Meconium Ileus, G: Gastrostomy.

Abbreviations at Column “Associated findings and features” (Ass.):

FH: Fetal Hydrops, Int. & Vent.: The patient was intubated and mechanical ventilation was started, FA: Fetal Anemia, BT: Blood transfusion, MSAF: Meconium stained amniotic fluid, HP: Hemoperitonium, RD: Respiratory distress, SBS: Short bowel syndrome, TPN: Total parenteral nutrition, Asc.: Ascites, MP: Meconium peritonitis, E: Enterostomy, CF: Cystic Fibrosis, MI: Meconium Ileus, MR: Mental Retardation, Hct: Hematocrit, Hb: Hemoglobin, NB: Necrotic bowel, DIC: Disseminated intravascular coagulopathy, TRAP: Twin reverse arterial perfusion.

Abbreviations at Column “Outcome” (O):

G: Good, B: Bad, ID: Intrauterine Death, PT: Pregnancy terminated, Mis: Miscarriage

Other Abbreviations:

N: normal, wk: weeks, d:days, g: gram, max: Maximum, min: Minimum, abd.: abdomen.
